# Polybenzyl Glutamate Biocompatible Scaffold Promotes the Efficiency of Retinal Differentiation toward Retinal Ganglion Cell Lineage from Human-Induced Pluripotent Stem Cells

**DOI:** 10.3390/ijms20010178

**Published:** 2019-01-05

**Authors:** Ta-Ching Chen, Pin-Yi She, Dong Feng Chen, Jui-Hsien Lu, Chang-Hao Yang, Ding-Siang Huang, Pao-Yang Chen, Chen-Yu Lu, Kin-Sang Cho, Hsin-Fu Chen, Wei-Fang Su

**Affiliations:** 1 Department of Ophthalmology, College of Medicine, National Taiwan University, Taipei 10002, Taiwan; tachingchen@gmail.com (T.-C.C.); chyangoph@ntu.edu.tw (C.-H.Y.); sean.maxen@gmail.com (D.-S.H.); 2 Graduate Institute of Clinical Medicine, College of Medicine, National Taiwan University, Taipei 10002, Taiwan; 3 Molecular Imaging Center, National Taiwan University, Taipei 10617, Taiwan; 4 Graduate Institute of Medical Genomics and Proteomics, College of Medicine, National Taiwan University, Taipei 10002, Taiwan; r02424027@ntu.edu.tw (P.-Y.S.); jllsh99@hotmail.com (C.-Y.L.); 5 Schepens Eye Research Institute, Massachusetts Eye and Ear, Harvard Medical School, Boston, MA 02114, USA; Dongfeng_Chen@MEEI.HARVARD.EDU (D.F.C.); Kinsang_Cho@MEEI.HARVARD.EDU (K.-S.C.); 6 Boston VA Healthcare System, Boston, MA 02130, USA; 7 Institute of Plant and Microbial Biology, Academia Sinica, Taipei 11529, Taiwan; ritalu@gate.sinica.edu.tw (J.-H.L.); paoyang@gate.sinica.edu.tw (P.-Y.C.); 8 Geriatric Research Education and Clinical Center, Office of Research and Development, Edith Nourse Rogers Memorial Veterans Hospital, Bedford, MA 01730, USA; 9 Division of Reproductive Endocrinology and Infertility, Department of Obstetrics and Gynecology, College of Medicine and the Hospital, National Taiwan University, Taipei 10002, Taiwan; 10 Department of Materials Science and Engineering, National Taiwan University, Taipei 10617, Taiwan

**Keywords:** induced pluripotent stem cells, retinal ganglion cells, optic neuropathy, glaucoma, polybenzyl glutamate, electrospinning scaffold, tissue engineering

## Abstract

Optic neuropathy is one of the leading causes of irreversible blindness caused by retinal ganglion cell (RGC) degeneration. The development of induced pluripotent stem cell (iPSC)-based therapy opens a therapeutic window for RGC degeneration, and tissue engineering may further promote the efficiency of differentiation process of iPSCs. The present study was designed to evaluate the effects of a novel biomimetic polybenzyl glutamate (PBG) scaffold on culturing iPSC-derived RGC progenitors. The iPSC-derived neural spheres cultured on PBG scaffold increased the differentiated retinal neurons and promoted the neurite outgrowth in the RGC progenitor layer. Additionally, iPSCs cultured on PBG scaffold formed the organoid-like structures compared to that of iPSCs cultured on cover glass within the same culture period. With RNA-seq, we found that cells of the PBG group were differentiated toward retinal lineage and may be related to the glutamate signaling pathway. Further ontological analysis and the gene network analysis showed that the differentially expressed genes between cells of the PBG group and the control group were mainly associated with neuronal differentiation, neuronal maturation, and more specifically, retinal differentiation and maturation. The novel electrospinning PBG scaffold is beneficial for culturing iPSC-derived RGC progenitors as well as retinal organoids. Cells cultured on PBG scaffold differentiate effectively and shorten the process of RGC differentiation compared to that of cells cultured on coverslip. The new culture system may be helpful in future disease modeling, pharmacological screening, autologous transplantation, as well as narrowing the gap to clinical application.

## 1. Introduction

Optic neuropathy, characterized by retinal ganglion cell (RGC) degeneration, is one of the leading causes of blindness all over the world [1], including optic neuritis, ischemic and traumatic optic neuropathies, glaucoma, and so on. Due to the lack of regenerative ability, RGC degeneration is a common feature of advanced optic neuropathy. Previous studies [2,3] have shown that rare RGCs extend their axons past the site of damage, and most of the RGSs die by following severe damage to the mouse optic nerve. This irreversible damage would cause blindness in affected humans. Therefore, developing an effective approach to rescue injured or dying RGCs and to promote cellular or axonal regeneration is an urgent need for these patients.

In recent years, some neuroprotective approaches which demonstrated neuroprotective effects in animal models have shown limited efficacy for RGC regeneration in clinical studies [4,5,6]. Now, the development of stem cell therapy further provides us a new perspective in this field. Retinal cells, including retinal pigment epithelium (RPE), photoreceptors, and RGCs, have caught great attention in the study of stem cell biology and therapeutics for years [7,8,9,10,11]. Several sources of stem and progenitor cells have been discussed in treatment of optic neuropathy [12,13]. Among them, induced pluripotent stem cells (iPSCs) are a specific stem cell type induced from adult somatic cells through reprogramming by transduction of defined transcription factors [14,15]. iPSCs share the same features as embryonic stem cells in morphology, proliferative ability, surface antigens, gene expressions, epigenetic status of pluripotent cell-specific genes, and telomerase activity [15,16]. Furthermore, iPSCs can be derived from the patient’s own somatic cells, the so-called patient-specific iPSCs, to avoid potential adverse cellular reactions, including immune rejection after transplantation of the iPSC derivatives. For retinal neurons, iPSCs can not only differentiate into a single type cell but also develop a 3D retinal primordium, reported as a retinal organoid from iPSCs in a culture dish [17,18]. In these previous studies, the neurites of RGCs that differentiated from iPSCs exhibited limited ability to extend. However, one key characteristic of functional RGCs is the long axons in a highly organized manner for transmitting visual information to the brain. We therefore need novel approaches to have iPSC-derived RGCs with long axons that could be directionally guided. 

Bioengineering approaches potentially offer unique advantages and game-changing solutions to overcome the current limitations. Advances in material science have enabled unprecedented control over the biochemical and biophysical properties of materials [19,20,21,22]. Electrospinning is a technique to fabricate nanofibrous scaffold that is a biomimetic of the hierarchical structure of extracellular matrix and can provide high surface area with interconnected pores [23]. It has been proven that axons of RGCs can also be guided through tissue engineering approaches, such as projecting axons along the fibrous scaffold and the gradient of guidance cues [24,25]. With the electrospinning process, one can generate biomaterials that are tuned to create an artificial niche to both efficiently differentiate iPSCs into mature neurons and enhance nerve growth/regeneration [26,27,28,29]. 

In the present study, we proposed the polybenzyl glutamate (PBG) as the substrate of biocompatible scaffold to promote the growth and differentiation of RGC progenitors. PBG is a peptide-based polymer, and it has been demonstrated that polypeptide is biocompatible without negative immune response [30,31]. Furthermore, PBG is fabricated into 3D scaffold through electrospinning technique and contains glutamate that is known as a neuron stimulant [32]. Recently, our team has reported that PC-12 cells cultured on PBG scaffold demonstrated excellent neurite outgrowth [23]. Now, we examine the effect of PBG scaffold on iPSCs-derived neural progenitors. To the best of our knowledge, this is the first report to apply PBG biocompatible scaffold in promoting cultures of neural stem cells as well as RGC progenitors. 

## 2. Results

### 2.1. Induction of hiPSC Differentiation to RGC-Like Cells on PBG Scaffold

**Figure 1:**
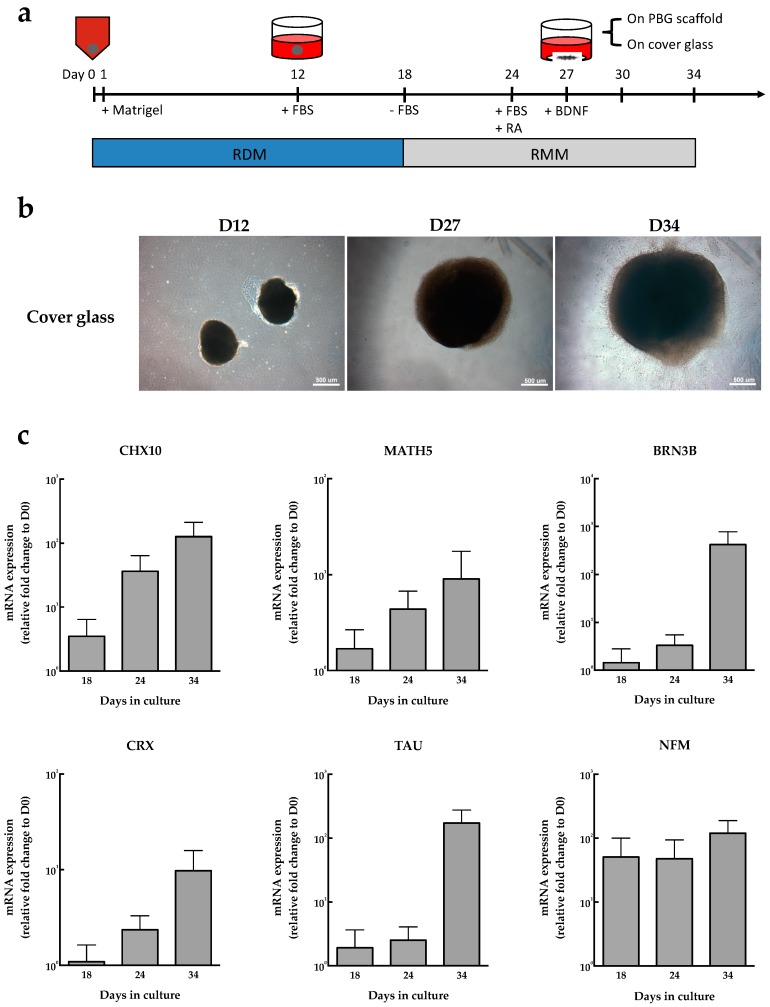
Induction of human-induced pluripotent stem cell (hiPSC) differentiation to RGC-like cells. (**a**) The flow chart of culture procedure of hiPSC-derived RGC-like cells. In brief, the hiPSCs were dissociated to single cells, and reaggregated to develop into embryoid bodies (EBs) in retinal differentiation medium (RDM) in V-bottomed low cell adhesion 96-well plate on Day 0, followed by adding 0.5% Matrigel on Day 1–18 and 1% FBS on Day 12–18. On Day 18, the culture condition was changed to retinal maturation medium (RMM), followed by addition of 1% FBS and 0.5 μM retinoic acid in RMM on Day 24, and then the aggregates placed into adherent culture on Day 27 with RMM containing 100 ng/mL BDNF. (**b**) In vitro time-course images of neural spheres cultured on cover glass. Scale bar = 500 μm. (**c**) The mRNA expression of RGC-associated genes at different time points of culture period. The relative mRNA expression of *CHX10*, *MATH5*, *BRN3B*, *CRX*, *TAU*, and *NFM* in hiPSC-derived RGC-like cells were analyzed on Day 18, Day 24, and Day 34, respectively.

To generate RGC-like cells from human-induced pluripotent stem cells (hiPSCs), we used a culture method modified from previous studies [18,33] (Figure 1a); Figure 1b demonstrates the morphology of neural spheres in different stages. We further examined retinal neuron differentiation under this culture condition by assessing the expression of retinal progenitor and RGC specific markers of the cell clumps. As we know, in human retinogenesis, *CHX10* is expressed in retinal progenitor cells and *CHX10* expression is lost after differentiation of progenitor cells except for bipolar cells [34]. It is implied that *CHX10* may play an important role for differentiation in all retinal progenitor cells [35]. The present data showed that the expression of *CHX10* increased rapidly in early stage and kept in high level until Day 34 (Figure 1c), suggesting that many differentiated hiPSCs were at the stage of retinal progenitor cells before Day 34. Formation of RGCs was regulated by *MATH5* and *BRN3B*, and it has been reported that *Math3* and *Brn3b* double null mice exhibited loss of RGCs during development [36], suggesting that transcription factors *MATH5* and *BRN3B* are crucial to determine the RGC formation and differentiation during development. As shown in Figure 1c, the expressions of *MATH5* and *BRN3B* were dramatically increased during the cell culture period. *CRX* is a photoreceptor-specific transcription factor and essential for maintenance of mammalian photoreceptors [37,38]. In our experiments, *CRX* expression was also up regulated until Day 34. We further investigated the expressions of axonal markers *TAU* and *NFM*, which are associated with the structural components of neurons. Our data showed that *TAU* expression was dramatically increased on Day 34, and the expression of *NFM* exhibited a relatively high level during the entire culture period. Collectively, the differentiation of RGC lineage could be induced from hiPSCs by following the present induction protocol.

**Figure 2:**
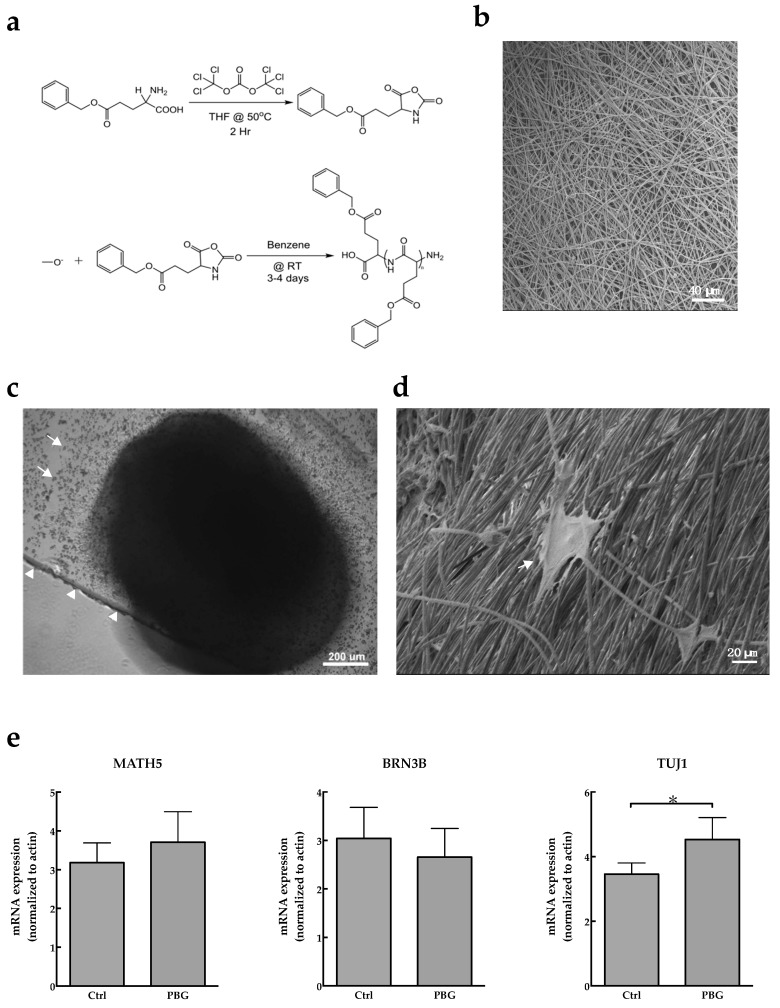
Induction of hiPSC differentiation to RGC-like cells on polybenzyl glutamate (PBG) scaffold. (**a**) Synthetic scheme of polyglutamate of PBG. (**b**) Scanning electron microscope (SEM) image of PBG scaffold. Scale bar = 40 μm. (**c**) The image of iPSC-derived RGC-like cells cultured on the PBG scaffold (arrow) was recorded by phase contrast microscope on Day 34. The arrowheads indicate the edge of glass cover. Scale bar = 200 μm. (**d**) SEM image of hiPSC-derived RGC-like cells (arrow) on the PBG scaffold, and the neurite was adhesive grew on the PBG scaffold. Scale bar = 20 μm. (**e**) The mRNA expression of *MATH5*, *BRN3B*, and *TUJ1* in hiPSC-derived RGC-like cells cultured on cover glass (Ctrl) or PBG scaffold (PBG) on Day 34. Values are presented as mean ± SEM, *n* = 3, * *p* < 0.05.

In order to investigate the effects of PBG scaffold on differentiation of hiPSCs, the aggregates were adherently cultured on PBG scaffold coated with 3% Matrigel in RMM with 100 ng/mL BDNF on Day 27. The chemical structures of PBG are shown in Figure 2a, and the microscopic morphology of PBG scaffold is shown in Figure 2b. HiPSCs were adhesive cultured on PBS scaffold (Figure 2c), and it demonstrates that hiPSCs were already seeded on PBG scaffold and grew with long neurites on Day 34 by using electron microscopy (Figure 2d). We observed that neurites extended along the PBG fiber on scaffold, implicating its potential to drive axon guidance. Furthermore, the mRNA expression that cells cultured on the cover glass or PBG scaffold was investigated (Figure 2e). The expressions of *MATH5* and *BRN3B* of PBG group were not increased compared to that of control group. However, the mRNA expression of *TUJ1*—the major component of microtubules and also used as a neuronal marker—was significantly increased in the PBG group (*p* = 0.041).

### 2.2. Determination of RGC Lineages and Axon Growth of hiPSC-Derived RGC-Like Cells

**Figure 3:**
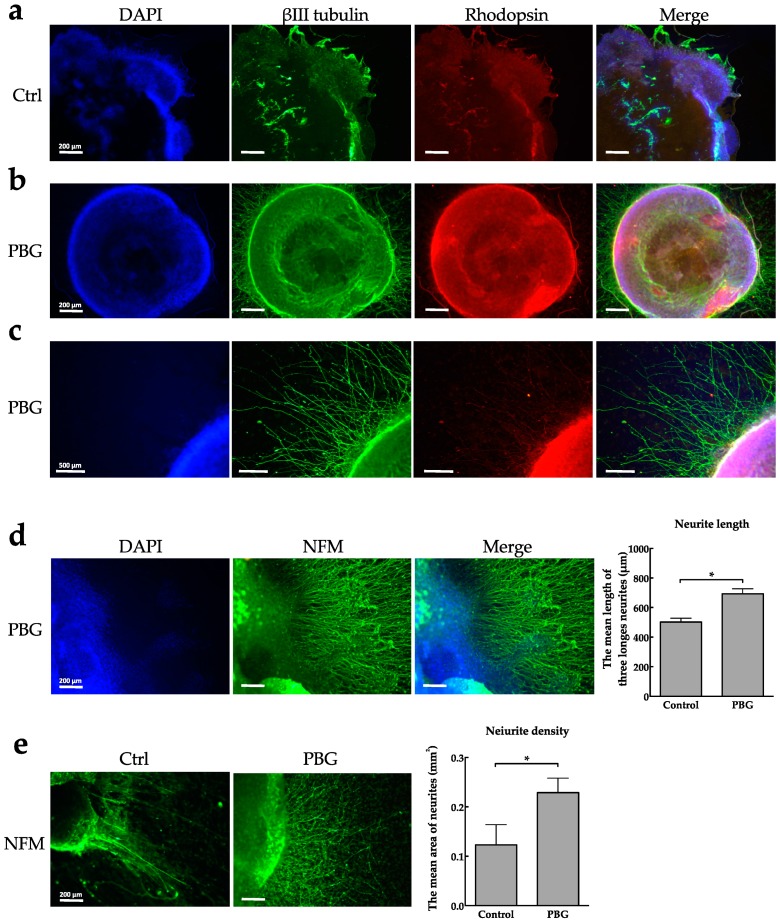
PBG scaffold promotes the differentiation of hiPSC toward RGC-like cells. (**a**–**c**) Representative images of hiPSC-derived RGC-like cells cultured on cover glass (**a**) and PBG scaffold (**b**,**c**), and cells were stained with DAPI and anti-βIII tubulin and anti-rhodopsin antibody. (**d**) The neurites of hiPSC-derived RGC-like cells were labeled by immunofluorescence with anti-NFM antibody, and the neurite length of hiPSC-derived RGC-like cells cultured on the cover glass or PBG scaffold on Day 34 was measured in the right panel. Values are presented as mean ± SEM, *n* = 9, * *p* < 0.05. (**e**) The neurites of hiPSC-derived RGC-like cells were labeled by immunofluorescence with anti-NFM antibody, and the neurite density of hiPSC-derived RGC-like cells cultured on the cover glass or PBG scaffold on Day 34 was measured in the right panel. Scale bars = 200 or 500 μm. Values are presented as mean ± SEM, *n* = 3, * *p* < 0.05.

After adherent culture on a PBG scaffold (Figure 3b) or coverslip (as control, Figure 3a) for three days, the early optic vesicle (OV)-like structure, which is the first step of eye development [39], was more easily observed in the PBG group than the control group. In the outer and inner shells, there was strong expression of βIII tubulin displayed in bright green fluorescence (Figure 3b). As we know, βIII tubulin is a neural specific type of tubulin that is expressed in microtubules of the cytoskeleton. However, it has been reported that βIII tubulin is also expressed in the developing nerve fiber layer, and highly expressed in RGCs likely because of its neuronal origin [40]. Furthermore, neurites grew prominently from the margin of the OV-like structure on PBG scaffold (Figure 3c). Rodopsin, a reliable marker of rod photoreceptor cells, was localized below the outer shell (Figure 3b). This indicates that hiPSC differentiation toward photoreceptor precursors is promoted. These morphologic characteristics described above were much less obvious in neural spheres cultured on the control group (coverslip) (Figure 3a). The neurites that extended from the edge of the OV-like structure were further confirmed by their expression of neurofilament medium (NFM) (Figure 3d,e). The neurite length and density were significantly increased in cultures grown on a PBG scaffold compared to that of the control group, indicating that PBG scaffold promote neurite outgrowth of hiPSC-derived RGC-like cells in vitro. 

### 2.3. RNA-seq Analysis of iPSC-Derived RGC-Like Cells Cultured on PBG Scaffold

**Figure 4:**
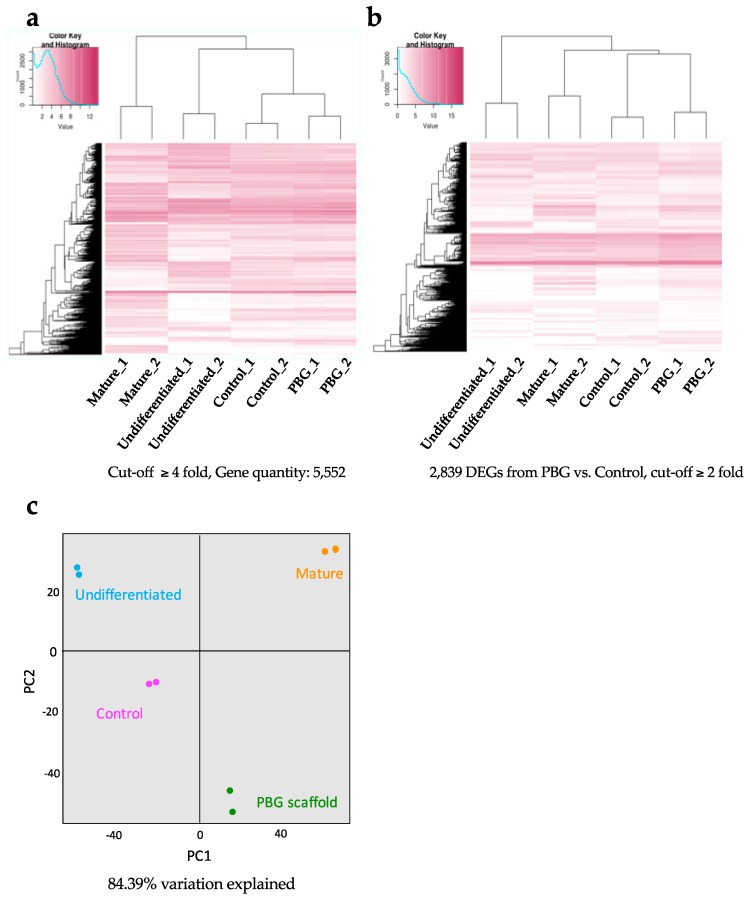
The transcriptome differences between hiPSC-derived RGC-like cells cultured on the cover glass and PBG scaffold on Day 34. (**a**) RNA-seq is performed in duplicate in each group (undifferentiated, mature, control, and PBG scaffold), and the expression profiles (5552 genes) of each group show consistent changes, which are illustrated by heat map. The relative expression levels are presented by using a color key. (**b**) Differentially expressed genes were identified between the PBG scaffold group and control group from RNA-seq analysis (2839 genes, fold change ≥2, adjusted *p*-value < 0.05). Global gene expression patterns of differentially expressed genes between four groups are shown by heat map. The relative expression levels are presented by using a color key. (**c**) Two-thousand-eight-hundred-and-nine differentially expressed genes from hiPSC-derived RGC-like cells cultured on cover glass (control) and PBG scaffold are analyzed by principal components analysis. PC1 and PC2 that represent 84.39% of variability are plotted on the x- and y-axis, respectively.

To further explore the molecular mechanisms and define the gene expression profiles, we performed RNA-seq analysis to investigate the transcriptomes of hiPSC-derived RGC-like cells cultured on coverslip and PBG scaffold on Day 34. These data were compared with the transcriptome of undifferentiated hiPSCs (downloaded from GSE73211) [41] as well as mature retinal neurons (downloaded from GSE40524) [42]. Global gene expression patterns disclosed 5552 genes showing highly variable expression patterns (cut-off fold change ≥4, total genes: 26,372) among the four groups (undifferentiated, mature, control, and PBG scaffold) (Figure 4a).

Subsequently, we identified 2839 differentially expressed genes (DEGs) between the PBG group and the control group (fold change ≥2, adjusted *p*-value < 0.05) from the total 26,372 genes (supplementary Table S1). Within these DEGs, we observed that the expression patterns of the PBG group and the control group are closer to mature retinal tissues than undifferentiated iPSCs (Figure 4b). In total, 1669 of which were upregulated and 1170 of which were downregulated in hiPSC-derived RGC-like cells that cultured on PBG scaffold, compared to that of in the control group. The principal component analysis is subjected to visualize the dissimilarity of transcriptomes by analyzing the 2839 DEGs among the four groups (Figure 4c). The data set was visualized along the first two principal components with 84.39% variation explained. We found that there were four distinct clusters, and along the most prominent axis—PC1—iPSC-derived RGC-like cells cultured on PBG scaffold on Day 34 were significantly different from the cells of the control group. Cells of the PBG group were clustered closer to the mature retinal neurons compared to cells of the control group along the PC1 axis. The tendency was observed when we just looked into the DEGs (Figure 4c). 

**Figure 5:**
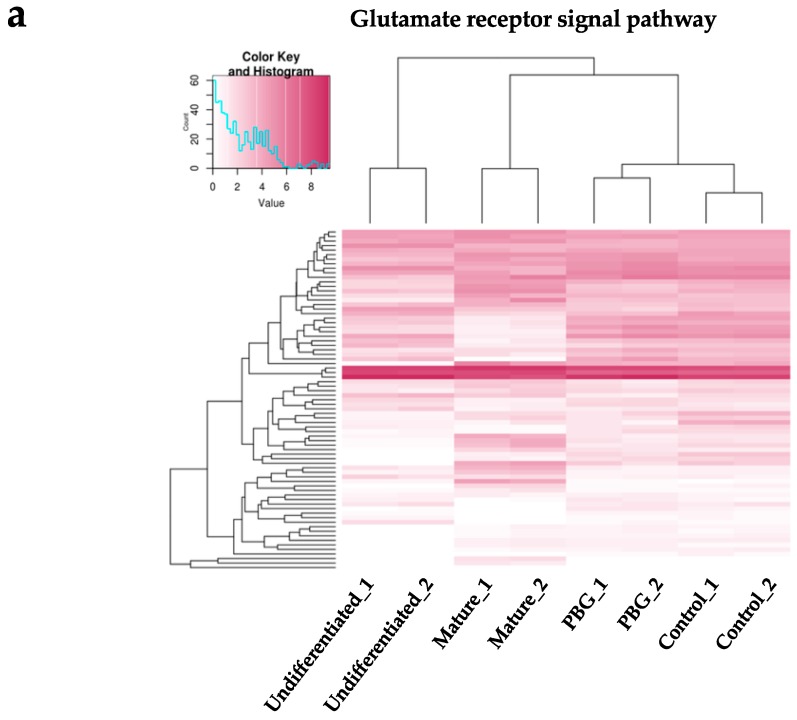

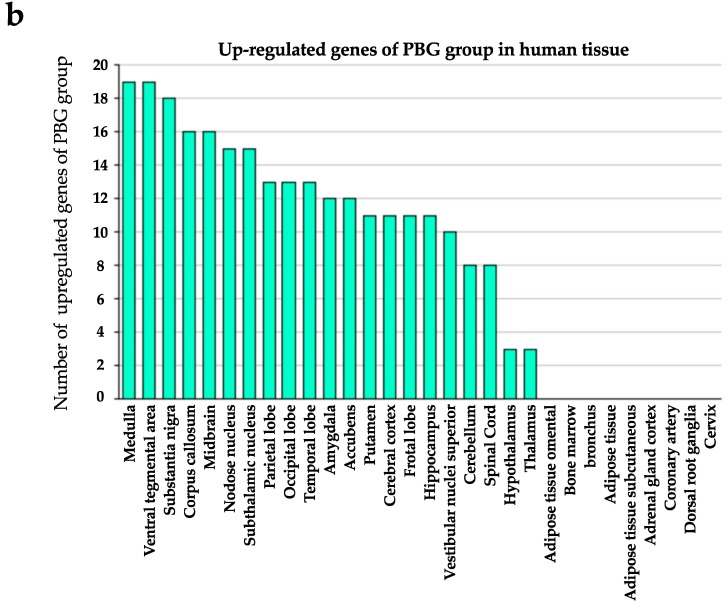
The transcriptome differences of glutamate mediated signal pathway related genes amongst four groups. (**a**) The transcriptional profiles of 75 genes related to the glutamate receptor signal pathway are shown as a heat map. The color key indicates gene expression values. (**b**) Upregulated genes of PBG group in central nerve system that analyzed by ExAtlas gene ontology analysis.

Since PBG scaffold contained glutamate, which is an excitatory neurotransmitter that plays the principal role in neural activation, we examined the glutamate receptor signal pathways to see if specific genes are involved in hiPSC-derived RGC-like cells cultured on PBG scaffold. We selected 75 genes closely related to the glutamate receptor signal pathway (GO:0007215) and the expression patterns among the four groups are shown in Figure 5a. Comparing to the overall change of gene expression of glutamate signaling-related genes among the four groups, more prominent changes of gene expression were observed in association with the glutamate-mediated pathway related genes (Figure 5a), suggesting that the glutamate receptor signal pathway may play an important role in hiPSC-derived RGC-like cells cultured on the PBG scaffold. Also, it revealed that the upregulated genes of PBG group were expressed in central nerve system by using ExAtlas (Figure 5b), suggesting that PBG scaffold may contribute to the differentiation from hiPSC to neurons. 

**Figure 6:**
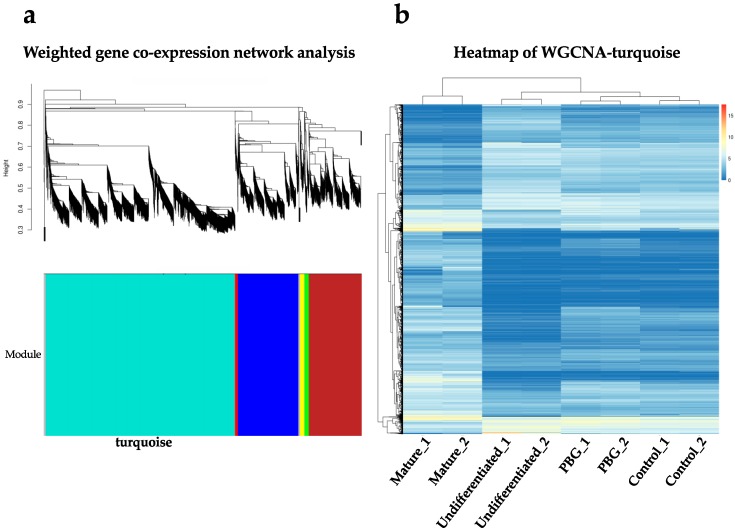
Weighted gene co-expression network analysis (WGCNA) analysis of correlation patterns among genes. (**a**) Hierarchical clustering dendrogram of genes that dissimilarity based on topological overlap. (**b**) Heatmap of turquoise module analyzed by WGCNA.

Subsequently, we identified the co-expression gene network modules among these transcriptome data in order to pin down specific biological functions. The RNA-seq data were subjected to weighted gene co-expression network analysis (WGCNA). As a result, we identified three major gene modules—turquoise, blue, and brown—which are co-expressed among these transcriptome data sets. These three modules cover 3895, 1249, and 1082 genes, respectively (Figure 6a). When we looked into the main gene module (turquoise), it contained genes that are highly associated with phototransduction (GO:0007602), photoreceptor cell development (GO:0042462) and differentiation (GO:0046530), retinol metabolic process (GO:0042572), eye development (GO:0001654) and morphogenesis (GO:0048592), and retina development (GO:0060041) (Table 1). The heat map of the turquoise module is shown in Figure 6b, showing the distinct expression patterns in this gene modules among the samples. 

## 3. Discussion

Optic neuropathy, including advanced glaucoma, has been regarded the major cause of blindness all over the world, and it has been reported that there will be 79.6 million people with glaucoma in 2020 [1]. Stem cell-based therapies offer a treatment option for patients of blindness due to ocular neurodegeneration [43,44]. Currently, stem cell-based therapies have been appraised to replace dysfunctional or dying retinal cells, such as RPE, photoreceptors, and RGCs. However, transplantation of RGCs is more challenging than transplanting RPE because functional RGCs need a delicate topography and long axons pass through optic disc for transmitting visual information to brain to form our vision [45,46], and it is urgent need in clinical practice to obtain RGCs with long axons in a highly organized manner for transplantation in vitro. In this regard, a series challenge of stem cell-based therapy for RGC is to obtain enough RGC progenitors that have long axon and then are transplanted adequately and functionally. 

Although it has reported that differentiation of stem cells to RGCs could be achieved by cultivation with a 2D adherent culture [10,47,48], using a CRISPR-engineered reporter cell line with adherent culture [49], isolated from 3D retinal aggregates [50,51], or combining cultivation of 3D floating aggregates with a subsequent 2D adherent culture [18,52]. These approaches address their efforts to acquire RGCs with adequate features, and multiple RGC markers are usually used to represent differentiated RGCs. Tanaka and his colleagues reported that iPSC-derived cells were defined as RGC lineage by upregulation of *BRN3B*, *MATH5*, *ISLET1*, *SNCG*, and *TUJ1* [18]. In addition, Kobayashi et al. reported that iPSC-derived RGCs were characterized using RGC markers in a 120-day differentiation protocol, including *ATOH7*, *BRN3B*, *ISL1*, *RBPMS*, and *THY1* [51]. However, RGCs with directed neurites/axon that are benefit to clinical use are not reported in these approaches. In this regard, we demonstrate the ability to massively obtain hiPSC-derived RGC progenitors that cells grew on PBG biocompatible scaffold. PBG is a peptide-based polymer which biomimics the structure of the extracellular membrane. The PBG contains glutamate that could promote signal transport between presynaptic and postsynaptic cells and activate the downstream signaling pathway involved in cell survival, metabolism, and proliferation [53]. In this study, we observed that the neurites of hiPSC-derived RGC progenitors could grow along with the fibers of PBG scaffold (Figure 2d). It may imply that PBG scaffold could support for the cells to adhere and grow. Since the scaffold contains glutamate, a neural stimulant, we are curious about the possible biochemical effects.

Biomaterials, such as biocompatible scaffolds, have been used in the field of tissue engineering for regeneration medicine [54]. These scaffolds are made to have molecular modifications and serve as substrate that can promote cell proliferation and differentiation to reconstruct damaged tissue or organ [21]. It has reported that cell survival of RGCs isolated from mice was increased by growing on a polylactic (PLA) scaffold [25], and the axons of RGCs were navigated by using immobilized guidance cue on PLA scaffold [24]. It is also reported that an injectable biomimetic scaffold was used for growing primary rat RGCs, and this polymer scaffold provide mechanical and chemical environment to support RGC growth by formation a laminar sheet-like structure and containing L1 integrin binding sites (arginine-glycine-aspartic acid, RGD) [55]. Different from PLA scaffold, we differentiated hiPSCs to RGC-like cells on PBG scaffold so that it is beneficial for clinical application. Also, the PBG is synthesized to contain glutamate and fabricated into three-dimensional (3D) structured polymer scaffold through electrospinning technique, meaning that the PBG scaffold with physical adherence property can not only support for the cells to adhere, but influence the growth of cells with its chemical characteristics. Our team has proved that PBG scaffold could promote the neurite outgrowth in differentiated PC-12 cells that induced by nerve growth factor, and neurite outgrowth is increased with increasing amount of glutamate release in the scaffold [23]. In this study, the OV-like structure is formed on D30 in the PBG group (Figure 3b), and the well-extended neurites grew radially outward from the clump (Figure 3b,c). It indicates that the PBG scaffold serving a culture substrate can promote the differentiation of hiPSC toward retinal lineage. On the other hand, it is potent to accelerate the process of RGC differentiation from iPSCs by following the optimization culture protocol in vitro. However, RGC differentiation from hiPSCs is still inefficiency [56]. In our experiment, hiPSCs cultured on the PBG scaffold formed the OV-like structure on D30, but hiPSCs cultured on the glass coverslip did not form an OV-like structure in the same culture period (D30) (Figure 3a), indicating that PBG scaffold could shorten the culture period.

With immunostaining, we found that cells of the PBG group showed a stronger expression of βIII-tubulin, the RGC marker (Figure 3). In addition to differentiation toward RGC lineage, the expression of rhodopsin, a marker of rod photoreceptor cells, also increased beneath the outer shell that cells were cultured on PBG scaffold (Figure 3b). Simultaneously, it revealed that the upregulated genes of PBG group were expressed in the central nerve system in ontological analysis (Figure 5b), implying that PBG scaffold may promote hiPSC differentiation toward neuronal lineage. These data indicate that hiPSCs grew on the PBG scaffold exhibit higher efficiency of neuronal differentiation from stem cells toward RGC lineage or, more generally, retinal lineage, compared to that of hiPSCs grew on the glass coverslip. 

In our team’s previous work [23], we demonstrated that the PBG scaffold is biodegradable and proved that part of the scaffold slowly resolved into solution. Furthermore, it is also demonstrated that materials released from PBG did not trigger cell death by MTT assay. It is possible that trace amounts of glutamate will be released into our culture medium. As we know, glutamate is the major excitatory neurotransmitter in the retina, and its receptors have been shown to involve in normal visual activation of RGCs [57,58,59]. Therefore, we were curious to know if the culture difference is related to glutamate signaling pathway and we performed the RNA-seq analysis (Figure 5a). The results showed that the cells of the PBG group and the control group are closer to mature retinal tissue than undifferentiated iPSCs in the gene expression profiling of glutamate signaling pathway, indicating that cells in PBG group differentiated toward retinal lineage more efficiently. Combined with the results of our recent publication [23], we can reasonably suggest that release of glutamate from PBG scaffold may also play a role in promoting neurite outgrowth and neuronal maturation in iPSC-derived RGC progenitors. 

WGCNA is an approach to quantify the correlation of genes and cluster highly correlated genes into modules [60]. In this study, the differentially expressed genes were incorporated into six clusters of co-expressed by WGCNA analysis (Figure 6a). The first principal of modules was highly associated with neuronal differentiation: neuronal maturation. When we look into the module, the differentially expressed genes between four groups were mainly clustered with biological functions of eye development, retinal differentiation, and retinal metabolism (Table 1). Furthermore, the genetic profile of control and PBG group in the module was closed to mature retinal neurons compared to that of undifferentiated cells (Figure 6b). With these molecular evidence and morphological observations, hiPSC-derived RGC-like cells grown on PBG scaffold could form retinal organoid effectively and promote neuronal growth of RGC progenitors, suggesting that PBG scaffold accelerates the differentiation process compared to that of the control group.

Taken together, PBG scaffold promotes hiPSCs differentiate toward retinal lineage, and form an OV-like structure compared to the control group in the same culture period. Additionally, the hiPSC-derived RGC-like cells cultured on the PBG scaffold exhibit well-extended neurites grew radially outward from the clump. Further molecular evidence demonstrated that cells of the PBG group differentiate toward the retinal lineage and the effects of PBG scaffold on iPSC differentiation may be related to the action of glutamate signaling pathway. These findings are meaningful because of the following. First, the PBG scaffold could shorten the culture period since previous protocols take a longer period of time to have RGC progenitors from hiPSCs [56], indicating that PBG scaffold promotes hiPSC differentiation toward RGCs and retinal lineage effectively. Second, the neurite outgrowth of hiPSC-derived RGC progenitors is promoted and directed by PBG scaffold, suggesting that 3D structured PBG scaffold containing glutamate serves as a physical and chemical guidance cue of neurite alignment and outgrowth. Despite the current challenges of stem cell-base therapy, such as safety of transplantation and optimal timing for transplantation, the development of biomaterial scaffolds for stem cell differentiation is a promising modality for future research and clinical applications. We also recognized that there are limitations in our present study. Aggregated retinal progenitors were used for attachment to the scaffold and cultured. This method gave us a chance to observe the self-organization of OV-like structure, but also made it difficult to harvest pure RGC progenitors for further analysis such as RNA-seq. Therefore, the statistical difference between the PBG and control group could be underestimated.

We propose and demonstrate a novel biomaterial—the electrospinning PBG scaffold—for culturing the hiPSC-derived RGC progenitors as well as retinal organoids effectively. To the best of our knowledge, this is the first article describing the usage of PBG scaffold to promote the growth of RGC progenitors as well as neural stem cells. The new culture system has the potential in future disease modeling, pharmacological screening, and autologous transplantation, as well as narrowing the gap to clinical application.

## 4. Materials and Methods

### 4.1. Human iPSC (hiPSC) Culture

The culture of hiPSCs was based on previous reports [61]. In brief, the Gibco^®^ Human Episomal iPSC Line (Thermofisher, Rockford, IL, USA) was used in this study and maintained on a feeder layer of mouse embryonic fibroblasts (MEFs) in Primate ES medium (ReproCELL, Kanagawa, Japan) supplemented with 4 ng/mL of basic fibroblast growth factor (bFGF, Invitrogen, Carlsbad, CA, USA). hiPSCs were split every 7 days by mechanical methods with a 30-gauge insulin needle. The culture medium was changed daily in the undifferentiated state of hiPSCs.

### 4.2. Induction of hiPSC Differentiation to RGCs 

Induction of hiPSC differentiation employed a procedure based on SFEB methods [52]. In brief, hiPSCs were dissociated to single cells in Accutase (eBioscience, San Diego, CA, USA) and were resuspended in retinal differentiation medium (RDM, G-MEM supplemented with 20% KSR, 0.1 mM nonessential amino acids, 1 mM pyruvate, 0.1 mM 2-mercaptoethanol, 100 U/mL penicillin, and 100 μg/mL streptomycin) containing Y-27632 (20 μM; Merck Millipore, Darmstadt, Germany), IWR-1e (3 μM, Merck Millipore, Darmstadt, Germany), and 0.5% Matrigel (BD Bioscience, San Jose, CA, USA). After separation from the feeder cells by decantation (the feeder cells adhered to the gelatin-coated bottom of the dish), the floating hiPSCs collected from the medium were seeded into 10% Pluronic-coated V-bottomed 96-well plates (Costar, Cambridge, MA, USA) at 9000 cells per well. On day 12 (D12), the aggregates were transferred to 10% Pluronic-coated 24-well plates (Costar), and the medium was replaced with RDM containing 0.5% Matrigel and 1% FBS. On Day 15, CHIR99021 (3 μM, Merck Millipore, Darmstadt, Germany) and SAG (100 nM, Merck Millipore, Darmstadt, Germany) were added to the medium. On Day 18, the aggregates were transferred to retinal maturation medium (RMM, DMEM/F12 Glutamax medium containing the N_2_ supplement, 100 U/mL penicillin, and 100 mg/mL streptomycin) and were then cultured in the absence of FBS. Then we added retinoic acid (0.5 μM, Sigma, St. Louis, MO, USA) and 1%FBS on D24. The concentration of FBS was increased stepwise from 1% up to 10% over the course of the adhesion culture period. On Day 27, the adhesion culture started when the aggregates were transferred to 3% Matrigel-coated PBG scaffolds or cover glass in 24-well plates (Costar, Cambridge, MA, USA) in RMN medium containing FBS and 100 ng/mL BDNF (R&D Systems, Minneapolis, MN, USA) [18]. Cells were adhesive cultured until Day 34, and then cells were harvested for further observation.

### 4.3. Electrospun PBG Scaffolds

The preparation of electrospun PBG scaffolds was conducted according to the literature, with some modifications [23]. The PBG scaffold is fabricated from polypeptide containing glutamate; the PBG can be prepared through conventional chemical synthesis in large quantity and fabricated into 3D scaffold through electrospinning technique. The PBG material was synthesized first, and then the material was electrospun into a fibrous scaffold. All of the chemicals used in the synthesis were purchased from Aldrich Chemicals and used as received. Briefly, monomer γ-benzyl glutamate-N-carboxy anhydride (benzyl-glu-NCA) was synthesized by reacting L-glutamic acid γ-benzyl ester (5.00 g) and triphosgene (3.13 g) in 2:1 molar ratio in dry tetrahydrofuran (THF) at 50 °C for 2 h, and the PBG was synthesized by dissolving 5 g of the monomer benzyl-glu-NCA in 500 mL of anhydrous benzene and adding sodium methoxide initiator to the monomer solution in a molar ratio of 100:1. The reaction was carried out under dry nitrogen atmosphere at room temperature for 3 days. The fibrous PBG was precipitated out from the solution by adding methanol. The PBG was dried in vacuum at 40 °C. The weight average molecular weight of the PBG was determined in the range of 200 to 300 KDa by gel permeation chromatography (Breeze, Waters, Mildford, MA, USA). A 20 wt% polymer solution was prepared in a mixed solvent of THF and dimethyl acetate (DMAc) (wt. ratio of 9:1) for electrospinning. The spinning apparatus consists of a syringe pump (KDS-100; KD Scientific, Holliston, MA, USA), a power supply (You Shang Technical Corp., Fong-Shang, Taiwan), and a metal collector (Chuan Chi Trading Co. Ltd., Taipei, Taiwan). A rate of 5 mL/h at 20 kV was used to spin the fiber. The aligned fiber was collected on Al-foil covered on the metal drum running at a speed of 3200 rpm for 20 min. Fibrous membrane were cut into circular specimens, detached from aluminum sheets and placed into 24-well tissue culture plates (Costar, Cambridge, MA, USA). Fibers on coverslips were directly put into 24-well tissue culture plate. Culture plates with electrospun scaffolds were sterilized by ultraviolet light irradiation overnight and coated with 3% Matrigel (BD Bioscience, San Jose, CA, USA) in 4 °C overnight. Then the scaffolds were made ready for cell culture according to the procedure described in the literature [62]. 

### 4.4. Real-Time PCR

Total RNA was extracted from undifferentiated hiPSCs and differentiated hiPSCs using a RneasyMiniKit (Qiagen, Hilden, Germany). cDNA was synthesized with Maxima First Strand cDNA Synthesis Kit (Thermofisher, Waltham, MA, USA). Real-time PCR was performed using the 7500 Fast Real-Time PCR system (Applied Biosystems, Foster City, CA, USA). The primers and probes used in this study are listed in Table 2. Thermocycler conditions: an initial hold at 42 °C for 5 min; incubation at 95 °C for 10 s; and then 40 cycles at 95 °C for 5 s and 60 °C for 31 s. The relative quantities of targeted mRNA were assessed by evaluating threshold cycle (CT) values. Hypoxanthine phosphoribosyl transferase 1 (HPRT1) transcript expression was used as an internal control for quantification. The relative concentration of each the target genes was calculated using the ΔΔCT method [18].

### 4.5. Immunofluorescence Staining

Differentiated hiPSCs were harvested on D30 and then fixed with 4% paraformaldehyde (Sigma-Aldrich, St. Louis, MO, USA) for 15 min at room temperature. After three rinses with PBS, specimens were incubated with 0.1% Triton X-100 (Sigma-Aldrich) with 0.1% tween 20 for 15 min at room temperature and then washed three times with PBS for 5 min each, and then blocked with 2% BSA (Sigma-Aldrich) for 60 min at room temperature [63]. The following primary antibodies were used, mouse anti-human beta-III tubulin antibody (Millipore, Bedford, MA, USA), mouse anti-human Rhodopsin antibody (Millipore), and rabbit anti-human Neurofilament M (Millipore). Secondary antibody reactions were carried out by incubation with the corresponding species-specific Alexa Fluor-488-conjugated antibodies (Epitomics, Burlingame, CA, USA) for 40 min at room temperature in the dark. The cells were counterstained with Hoechst 33342 for nuclear staining. After four washes with PBS for 5 min each, specimens were mounted with Vectashield (Vector Laboratories, Burlingame, CA, USA). Finally, specimens were mounted in an antifade mounting medium (with DAPI) and observed under a fluorescence microscope (IX71, Olympus, Tokyo, Japan).

### 4.6. Measurement of Neurites

The length of the neurites was measured from the images taken at Day 34 after culturing and analyzed with ImageJ. The five longest neurites from each optic vesicle were chosen and traced from the edge to the end. After the calculation, the lengths of the three longest neurites were statistically evaluated [50]. The neurite density was measured by ImageJ. Cells were against to anti-NFM antibody, and the fluorescent images of hiPSC-derived RGC-like cells that incubated on cover glass or PBG scaffold were obtained on Day 34. In brief, the images were converted to grayscale, and the total area of neurites was calculated by ImageJ.

### 4.7. SEM Observation

The fibrous structure of electrospun scaffolds and cell morphology were characterized by scanning electron microscope (SEM, JEOL; JSM-6700F, Tokyo, Japan). At each time point, specimens were washed with PBS and fixed with 4% PFA for 2 days at 4 °C. Specimens were then post fixed in ½ strength Karnovsky’s fixative, dried by critical point dehydration, and observed under SEM [62].

### 4.8. Whole Transcriptome Analysis by RNA-Seq

Total RNA was extracted using the RNeasy kit and treated with DNase 1 (Qiagen, Hilden, Germany). Triplicate sets of RNA samples were obtained from Day 34 hiPSC-RGCs in both control group and PBG group. Whole transcriptome libraries generation and rRNA depletion were performed by the Australian Genomic Research Facility. Pair-end sequencing (100 bp) was performed using an Illumina HiSeq 2000. Bioinformatic analysis was performed using ExAtlas. Our transcriptome data was further compared with transcriptome data of undifferentiated iPSCs [41] as well as mature human retinal tissues [42].

### 4.9. Weighted Gene Co-Expression Network Analysis (WGCNA)

WGCNA was performed using the R package on transcriptome data following the standard method described on the authors’ website [60]. This unsupervised and unbiased analysis identifies distinct co-expression gene modules by clustering transcripts with similar expression patterns across samples. Transcriptionally variable genes with at least 4-fold changes between samples in the analysis were selected for the analysis. The genes were hierarchically clustered based on a variation measure of topological overlap matrix. The resulting dendrogram was used for module detection (minimum size 50; height cut-off 0.8). Gene modules were labeled in unique colors. Unassigned genes were labeled in gray.

### 4.10. Statistical Analysis

All data were expressed as the mean ± standard error of the mean (SEM). Differences between the means of experimental and respective control groups were calculated by Mann–Whitney U test. For data more than 3 groups, a Student’s *t*-test and analysis of variance (ANOVA) test followed by post hoc multiple comparisons were used. A *p*-value < 0.05 was considered statistically significant.

## 5. Conclusions

We propose and demonstrate a novel biomaterial, electrospinning PBG scaffold, for culturing the hiPSC-derived RGC progenitors as well as retinal organoids effectively. To the best of our knowledge, this is the first article describing the usage of PBG scaffold to promote the growth of RGC progenitors as well as neural stem cells. The new culture system has the potential in future disease modeling, pharmacological screening and autologous transplantation, as well as narrowing the gap to clinical application.

## Figures and Tables

**Table 1:** Transcriptome data set of turquoise.

Biological Process (Gene Ontology)	Fold Enrichment	FDR
Phototransduction (GO:0007602)	3.45	1.35 × 10^−4^
Photoreceptor cell development (GO:0042462)	3.16	7.53 × 10^−3^
Retinol metabolic process (GO:0042572)	3.08	1.29 × 10^−2^
Photoreceptor cell differentiation (GO:0046530)	2.66	4.59 × 10^−3^
Eye morphogenesis (GO:0048592)	1.94	4.53 × 10^−3^
Eye development (GO:0001654)	1.91	1.09 × 10^−6^
Retina development (GO:0060041)	1.83	2.72 × 10^−2^

FDR: False discovery rate.

**Table 2:** Primers for real time PCR.

Gene	Accession No.	Forward	Reverse
*Hprt1*	NM_000194.2	GGCAGTATAATCCAAAGATGGTCAA	GTCAAGGGCATATCCTACAACAAAC
*Chx10*	NM_182894.2	AACCCAATCTGGCTGGTAAATGA	CAGCAGGCCCTTAATGCGTA
*Brn3b*	NM_004575.2	TGACACATGAGCGCTCTCACTTAC	ACCAAGTGGCAAATGCACCTA
*Crx*	NM_000554.4	ACCCTGATCTCTAGAGCCCACAA	CTTAATGTCCCAGAACCCAGCA
*Math5*	NM_145178.3	CCCTAAATTTGGGCAAGTGAAGA	CAAAGCAACTCACGTGCAATC
*Tuj1*	NM_006086.3	GGCCAAGGGTCACTACACG	GCAGTCGCAGTTTTCACACTC
*Tau*	NM_001123066.3	CCAAGTGTGGCTCATTAGGCA	CCAATCTTCGACTGGACTCTGT
*NFM*	NM_005382.2	ACAACCACGACCTCAGCAGCTA	ATGACGAGCCATTTCCCACTTT

## Data Availability

All sequencing data are deposited at the GEO repository under the accession number GSEXXXXX. The data of this study are available from first and corresponding authors upon reasonable request.

## Supplementary Materials

Supplementary materials can be found at http://www.mdpi.com/1422-0067/20/1/178/s1.
